# Community, facility and individual level impact of integrating mental health screening and treatment into the primary healthcare system in Sehore district, Madhya Pradesh, India

**DOI:** 10.1136/bmjgh-2018-001344

**Published:** 2019-05-19

**Authors:** Rahul Shidhaye, Emily Baron, Vaibhav Murhar, Sujit Rathod, Azaz Khan, Abhishek Singh, Sanjay Shrivastava, Shital Muke, Ritu Shrivastava, Crick Lund, Vikram Patel

**Affiliations:** 1 Center for Chronic Conditions and Injuries, Public Health Foundation of India, Gurugram, India; 2 Alan J Flisher Centre for Public Mental Health, Department of Psychiatry and Mental Health, University of Cape Town, Cape Town, South Africa; 3 PRIME Project, Sangath, Bhopal, India; 4 Department of Population Health, London School of Hygiene and Tropical Medicine, London, UK; 5 Centre for Global Mental Health, Health Service and Population Research Department, Institute of Psychiatry, Psychology and Neuroscience, King’s College London, London, UK; 6 Department of Global Health and Social Medicine, Harvard Medical School, Boston, Massachusetts, USA; 7 Sangath, Goa, India

**Keywords:** mental disorders, primary healthcare, programme evaluation, health services research, India

## Abstract

**Introduction:**

Programme for Improving Mental Health Care (PRIME) designed a comprehensive mental healthcare plan (MHCP) for Sehore district, Madhya Pradesh, India. The objective of this paper is to describe the findings of the district-level impact evaluation of the MHCP.

**Methods:**

Repeat community-based CS were conducted to measure change in population-level contact coverage for depression and alcohol use disorders (AUD), repeat FDS were conducted to assess change in detection and initiation of treatment for depression and AUD, and the effect of treatment on patient outcomes was assessed using disorder-specific prospective cohort studies.

**Results:**

PRIME MHCP did not have any impact on contact coverage/treatment seeking for depression (14.8% at the baseline and 10.5% at the follow-up) and AUD (7.7% at the baseline and 7.3% at the follow-up) and had a small impact on detection and initiation of treatment for depression and AUD (9.7% for depression and 17.8% for AUD compared with 0% for both at the baseline) in the health facilities. Patients with depression who received care as part of the MHCP had higher rates of response (52.2% in the treatment group vs 26.9% in the comparison/usual care group), early remission (70.2% in the treatment group vs 44.8% in the comparison/usual care group) and recovery (56.1% in the treatment group vs 28.5% in the comparison/usual care group), but there was no impact of treatment on their functioning.

**Conclusions:**

While dedicated human resources (eg, Case Managers) and dedicated space for mental health clinics (eg, Mann-Kaksha) strengthen the ‘formal’ healthcare platform, without substantial additional investments in staff, such as Community Health Workers/Accredited Social Health Activists to improve community level processes and provision of community-based continuing care to patients, we are unlikely to see major changes in coverage or clinical outcomes.

**Key questions**

**What is already known?**

- World Health Organization’s mhGAP (Mental Health Gap Action Programme) guidelines have synthesized evidence-based interventions (e.g. psychotropic drugs, psycho-social interventions) to treat mental disorders. The key knowledge gap is about how to integrate these evidence-based interventions in primary care in low resource settings.
- For Improving Mental health carE (PRIME) designed a comprehensive district level Mental Health Care Plan (MHCP) to address this knowledge gap.

**What are the new findings?**

- PRIME MHCP did not have any impact on contact coverage for depression and AUD at the community level and a small impact on detection and initiation of treatment for depression and AUD in the health facilities. At the individual patient level, there was moderate impact of MHCP interventions.

**What do the new findings imply?**

- Establishing a collaborative model of care remains a very challenging task, especially in resource poor settings. However, facilitation by an implementation support team can play an important role in strengthening mental health services.
- Psychosocial therapies cannot be effectively delivered without engaging existing or new community-based resources.

## Introduction

In India, the weighted prevalence of any mental disorder is 10.6% (point prevalence) and 13.7% (lifetime prevalence) as per the findings of the National Mental Health Survey.^1^ Mental disorders account for 31 million Disability Adjusted Life Years, which is 13% of the total non-communicable disease burden in India.^2^ Unfortunately, the access to mental healthcare in India is very poor due to multiple demand and supply side barriers and this has led to a huge treatment gap for mental disorders. The treatment gap for common mental disorders is 85% and for severe mental disorders it is 73.6%.^1^

Three key policy initiatives viz., the National Mental Health Policy launched in 2014, the new National Health Policy^3^ and most importantly, the Mental Health Care Act,^4^ provide a unique opportunity to bridge this treatment gap in India.^4^ However, there are very few evidence-based implementation models in India which provide a plan to integrate mental healthcare delivery in existing primary healthcare system which can ultimately improve access to care. In this context, the work of the Programme for Improving Mental Health Care (PRIME) is a potential model for strengthening mental health services in India. PRIME began in 2011 as an implementation research effort to understand the best strategies for integrating evidence-based mental health interventions into the primary healthcare system, and includes five countries: Ethiopia, India, Nepal, South Africa and Uganda.^5^

In India, PRIME was implemented in the state of Madhya Pradesh. As part of the inception phase of PRIME, a draft district mental healthcare plan (MHCP) was developed (described below).^6^ A comprehensive, cross-country evaluation protocol was designed to assess the impact of the PRIME MHCP.^7^ In this paper, we describe the findings of the community, facility and individual level impact of the implementation of PRIME MHCP.

## Methods

### Setting

Sehore district in Madhya Pradesh was the implementation site for PRIME in India. It has a population of 1.3 million which is predominantly rural (81%) and the district covers an area of 6578 km^2^. Situational analysis identified a range of major health system challenges in developing and implementing MHCP in Sehore district. Poor governance, lack of sustainable mental health financing measures, severe shortages of skilled mental health professionals in the public health system, very few capacity building initiatives for general health professionals and non-specialists health workers, non-availability of essential psychotropic drugs, under-developed mental health information systems and low mental health literacy and stigma against people with mental disorders resulted in non-integration of mental health in primary care in Sehore district.^8^ During the first 2 years of the programme, a comprehensive MHCP for Sehore district was designed in partnership with the Department of Health Services, Government of Madhya Pradesh and other key stakeholders. This was followed by a pilot implementation in one community health centre (CHC) and refinement of the draft MHCP.

### Programme for Improving Mental Health Care mental healthcare plan

PRIME MHCP was implemented in three CHCs in Sehore district for a period of 25 months from 1 August 2014 till 31 August 2016. Initially, the plan was to implement MHCP in the entire Sehore district. However, during the inception phase, we realised that there are several health system and contextual challenges to programme implementation and that strong facilitation was necessary in order to instal, optimise and improve service delivery processes. Based on the human resource we had in the PRIME programme (one programme coordinator and six Case Managers), it was feasible to implement the programme in one subunit/block of the district which had three CHCs/Sub-District Hospitals. These CHCs provide services to 239 villages covering a population of 268 833.

PRIME MHCP consisted of service delivery and enabling packages. The enabling packages comprised cross-cutting interventions to ensure the smooth implementation of core mental health service delivery packages. There were three enabling packages: programme management, capacity building and community mobilisation. Programme management activities related to governance, human resource management and drug procurement and supply chain management started in March 2014. Training of Medical Officers, Front Line Workers and Case Managers was conducted from March to July 2014. A Health Management Information System was established in last week of July 2014 and regular process data were collected from 1 August 2014 until the end of the implementation phase (31 August 2016).

The four service delivery packages were awareness creation, identification, treatment and recovery for three priority mental disorders: depression (including maternal depression), psychosis and alcohol use disorders (AUD). Mental health services were delivered on healthcare platform and community platform.

Mental health Case Managers employed by PRIME played a very important role in implementation of MHCP. There was a total of six Case Managers, two (one male and one female) Case Managers in each of the CHC. A room was allocated in each CHC for provision of mental health services. This room was named as ‘Mann-Kaksha’ (translated as ‘Mental Health Corner’ in English) and was managed by the Case Managers. Case Managers contacted patients attending outpatient clinics and if their presenting complaints were similar to those mentioned in the master chart of the mental health Gap Action Programme (mhGAP) intervention guide, they screened the patients using Patient Health Questionnaire-9 item (PHQ-9) (for depression) and Alcohol Use Disorder Identification Test (AUDIT) (for AUD). Case Managers shared PHQ-9/AUDIT scores with the Medical Officer who made the diagnosis and took the decision to prescribe anti-depressants/other medications depending on the severity. Patients who received confirmed diagnosis of depression/AUD by the Medical Officer were offered counselling treatment in the form of Healthy Activity Programme (HAP, for depression) or Counselling for Alcohol Problems (CAP, for AUD). If they agreed, they were enrolled in the programme. Generally, patients with PHQ-9 >14 received both anti-depressants and counselling while those with PHQ-9 ≤14 received only counselling. HAP is a manualised psychological treatment based on behavioural activation and it includes (1) psycho-education, (2) behavioural assessment, (3) activity monitoring, (4) activity structuring and scheduling, (5) activation of social networks and (6) problem solving.^9^ CAP is a manualised psychological treatment based on motivational interviewing.^10^ Women attending antenatal/postnatal clinics were also screened by Case Managers and if found positive for depression, they were offered HAP. The District Mental Health Programme Psychiatrist visited each of the CHCs once a month to provide consultation for severe cases, especially those with psychosis. Patients with psychosis were primarily identified by Case Managers in the community (see below). For these patients (and their caregivers), Case Managers provided psychoeducation and facilitated consultation with the Psychiatrist. Case Managers maintained regular follow-up with enrolled patients.

In addition to these services which were offered on healthcare platform, Case Managers visited villages which were under their respective CHCs to provide services on the community platform. During their visit to a village, Case Managers would meet community health workers (CHWs) in that village (eg, Accredited Social Health Activist, ASHA) and based on their inputs, contacted individuals to assess them for priority mental disorders using the master chart of the mhGAP intervention guide. Case Managers provided Mental Health First Aid (MHFA)^11^ to these individuals and if they felt the need for further evaluation and management, a referral slip to visit the CHC was provided.

### Overview of the study design

The overall aim of PRIME MHCP was to improve demand for mental health services at the population/community level, reduce the ‘missed opportunity’ at the health facility level by improving detection of depression and alcohol use disorder (AUD) and provide evidence-based services to individuals with priority mental disorders (depression, AUD and psychosis).

Hence, in the evaluation plan to assess the impact of district MHCP, outcomes at three levels were considered, namely population, health facility and individual/patient level. Our approach was guided by Tanahashi framework (used to evaluate coverage of services in a public health programme), a Theory of Change map and the multidisciplinary methods embedded within this theory.^7^

The following study designs were employed to evaluate the above mentioned MHCP outcomes:

- Repeat community-based cross-sectional surveys (CS) to measure change in population-level contact coverage for depression and AUD.
- Repeat facility-based detection surveys (FDS) to assess change in detection and initiation of treatment for depression and AUD.
- Disorder-specific prospective cohort studies to assess the effect of treatment on patient outcomes (effective coverage).

In addition to this, a multilevel case study was also undertaken to evaluate the process of implementation. A summary of the process data relevant to study designs included in this paper are presented in the results sections.

**Figure 1:**
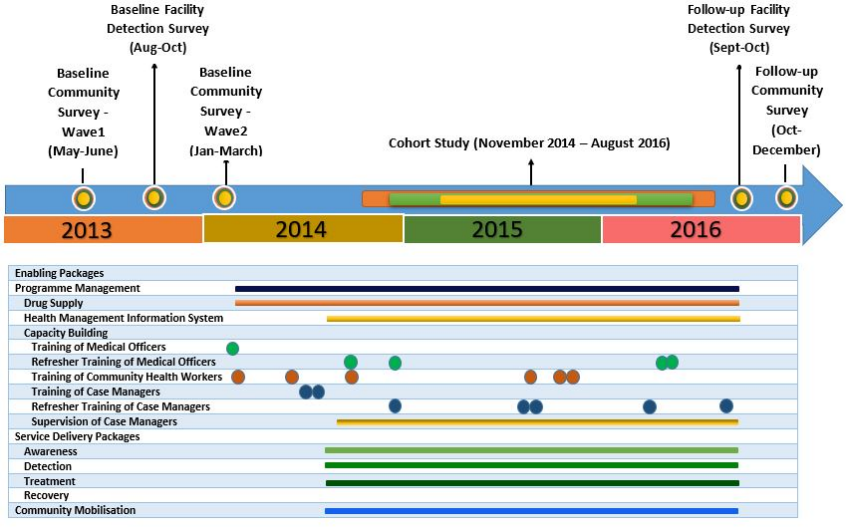
Programme for Improving Mental Health Care India mental healthcare plan timelines. FDS, facility-based detection surveys.

The timeline of baseline and follow-up CS and FDS, cohort study and implementation of PRIME MHCP is depicted in figure 1.

### Patient and public involvement

The development of the MHCP and the evaluation framework (including the research questions and outcome measures) was based on the Theory of Change workshops and qualitative data obtained during the inception phase. Key stakeholders such as service providers in the public health system, community members and patients were involved during this work in the inception phase.^6^ Community Advisory Board was established to provide feedback and inputs during the implementation phase, however, patients were not involved in the recruitment and conduct of the study. At the end of the programme, there is a plan to disseminate results to the members of the Community Advisory Board, patients and general community members in Sehore district.

The section below separately describes data collection methods, outcomes assessment and statistical analysis for community survey, facility detection survey and patient cohorts, respectively.

### Community survey

The baseline community survey was completed in 2013–2014 before the implementation of MHCP. The baseline prevalence and contact coverage for depression^12^ and AUD^13^ are reported in previous publications. The follow-up survey was conducted from October to December 2016, 26 months after the initiation of PRIME MHCP implementation.

#### Sample selection

There are 1031 villages and eight towns in Sehore district. The total population of Sehore district is 1 311 332 (rural: 1 062 870, urban: 248 462). After the completion of baseline community survey, the decision was taken to implement PRIME MHCP in only three CHCs as mentioned above. As a result, the sample for follow-up survey was selected from only those villages which were under PRIME CHCs. Of the total 239 villages which are officially covered by these three CHCs, effectively people from only 188 villages access health services from these CHCs and people from the rest of the 51 villages access services from Sehore town or Bhopal (capital of Madhya Pradesh) due to geographical proximity. Thus, the sample for follow-up survey was restricted to 188 villages (total population of 213 187) as this was the effective implementation area for community platform activities. From the baseline community survey, there were 1305 participants from 188 villages. In the follow-up community survey, the sample size was set to detect a change in contact coverage/treatment seeking from 7.7% (baseline) to 25% for AUD, which we considered was a meaningful increase from a population health perspective. As the prevalence and contact coverage/treatment seeking for AUD was almost half that of depression, the estimates were done for AUD so that we were assured sufficient power for the depression analyses. The sampling frame for follow-up survey comprised adults (18 years or old) selected from voter lists in 188 villages from the implementation area. The voter lists were obtained from the website of the Election Commission of India. The sample was selected from each of the voter lists. The number of individuals sampled per voter list was estimated according to the proportional contribution of the voter list to the total population. The first individual in the sample was randomly selected and then every nth individual was selected based on the sampling interval. Inclusion criteria for participation were fluency in spoken Hindi, residency in the selected household, time and ability to complete the full interview, willingness to provide informed consent, and absence of any cognitive impairment that was severe enough to interfere with the informed consent procedure or survey.

#### Data collection

The structured-interview schedule used for the follow-up survey was the same as the one used for the baseline survey (with some additional items related to travel time and costs to seek mental health services). Depression and AUD were measured using the PHQ-9 and AUDIT, respectively, which are validated and widely used in India.^14–16^ Participants who scored 10 or more on PHQ-9 were taken as probable cases of depression, participants who scored eight or more on AUDIT were taken to indicate probable cases of AUD. Probable cases were then asked questions related to seeking help for depression-related and AUD-related problems from specialist, generalist or non-formal providers in the past 12 months. Details about the treatment received, medications and costs of care (incurred by the individual) were elicited. Participants were then asked questions on suicidal ideation, adapted from the Mini-International Neuropsychiatric Interview, a semi-structured interview for the assessment of mental disorders. Sociodemographic factors assessed included possession of below poverty line card and employment with Mahatma Gandhi National Rural Employment Guarantee Act, both indicators of socioeconomic deprivation. The structured interview for the CS (as well as for the FDS and the cohort study) was administered using tablet computers linked to an online application programme (Mobenzi; www.mobenzi.com).

#### Outcomes

Our primary outcome was to measure change in contact coverage 26 months after the PRIME implementation began in August 2014, defined as the diﬀerence in the proportion of individuals with depression or AUD (PHQ-9 score ≥10 and AUDIT score ≥8, respectively) who sought treatment for their symptoms after the baseline survey.

#### Statistical analysis

The sociodemographic characteristics of the baseline survey population and the follow-up survey population were treated as categorical variables and compared with the χ^2^ test. Second, for both PHQ9-positive and AUDIT-positive participants, the number, proportion and 95% CI were reported for detection and for initiation of adequate evidence-based treatment. All analyses for the CS (as well as for the FDS and the cohort study) were conducted in Stata V.14.1^17^ and design-adjusted for the sampling design.

### Facility detection survey

The baseline FDS was conducted prior to implementation of the MHCP and the follow-up survey was completed from September to October 2016, 30 months after the first training of medical officers and 3 months after the last refresher training of medical officers.

#### Sample selection

Systematic random sampling was employed to collect a sample of patients exiting their clinical consultations in the three CHCs in the PRIME implementation area. The inclusion criteria for participation were the same as in the community survey, except that patients presenting with acute medical conditions were also excluded. Our sample size calculation was based on the assumption that the detection of depression and AUD would increase from 1% to 33%. With 90% power and level of significance of 5%, we needed a sample size of 33 patients with AUD. In order to obtain the required sample size of 33, we needed to approach 726 individuals. This was based on the assumption of 5% prevalence of AUD in primary care and 10% rejection rate for interviews. The sample size of 726 provided us with 95% power to detect change in detection of depression from 1% to 33% with the assumption of 10% prevalence of depression in primary care and 10% rejection rate.

#### Data collection

The structured interview consisted of sections pertaining to: sociodemographic characteristics; recent use of alcohol and tobacco; screening for AUD; screening for depression; details about the clinical consultation.

#### Outcomes

The outcome of interest was the estimation of the change in proportions receiving mental disorder-specific clinical services over time, including (1) diagnosis of depression or AUD by the primary healthcare worker and (2) initiation of minimal-adequate treatment plan by the primary healthcare worker. Ascertainment of the minimal-adequate treatment plan was based on the following criteria: (1) relevant advice (psychoeducation/psychological intervention) for reducing symptom severity; (2) an appropriate specialist referral; (3) an appropriate medication regimen; or (4) a combination of (1), (2) or (3). These outcomes were assessed by collecting information from the patient through a postconsultation interview that asked the patient whether they received a diagnosis, treatment plan or referral.

#### Statistical analysis

The sociodemographic characteristics of the baseline survey population and the follow-up survey population were treated as categorical variables and compared with the χ^2^ test. Proportion of patients with depression and AUD receiving diagnosis and initiation of treatment was estimated and 95% CIs were compared. All analyses were conducted in Stata V.14.1.^17^

### Cohort study

#### Sample selection

Recruitment into the depression, AUD and psychosis cohorts was initiated 3 months after the start of the MHCP implementation in Sehore district. During this time, initial training of staff and setting up of supervision and leadership processes were completed. This allowed for the services to run for several weeks before recruitment started. Participants were recruited from outpatient clinics in the three CHCs implementing the MHCP. Patients attending outpatient clinic were screened with the PHQ-9 and AUDIT by Case Managers, and were then assessed and diagnosed by medical officers. Patients who received either a diagnosis of depression or AUD and referred for treatment were recruited in the respective cohorts. The depression treatment cohort was further divided into two cohorts: general depression (non-perinatal population) and maternal depression (perinatal population). Women were considered perinatal if they were either pregnant or had a child younger than 1 year old. Treatment for severe mental disorders had priority over common disorders, so if patients were diagnosed with psychosis, patients were recruited into the psychosis cohort, regardless of their screening scores on the PHQ-9 and AUDIT. Not all patients attending outpatient clinic were screened by Case Managers due to time constraints. These patients, however, did consult the medical officer for their health complaints. Research team members then randomly screened these individuals in outpatient clinics after they completed consultation with the medical officer. Individuals who were not diagnosed by medical officers, but screened positive on PHQ-9 or AUDIT (by a research team member) were recruited as comparison groups (as they had not been diagnosed or referred to treatment). Participants in the comparison group received usual care for their general health complaints from the medical officer. They did not receive any mental health interventions. There were no perinatal women in the depression comparison group. The target sample size was set at 200 for depression and AUD cohorts, and 150 for the psychosis cohort. The details of the data collection and sample size calculation are provided elsewhere.^18^

#### Data collection

All participants were assessed three times: the baseline assessment was initiated at the clinic following enrolment into the cohorts (including the PHQ-9 and AUDIT), and finalised in the participants’ home (including demographic variables and the WHO Disability Assessment Schedule (WHODAS)). The time elapsed between the enrolment of patients and the completion of the baseline interview could not exceed 7 days, and was completed on average 3 days after enrolment. The first follow-up was conducted 3 months after recruitment (±2 weeks) for the depression, maternal depression and AUD cohorts, and 6 months (±2 weeks) after recruitment for the psychosis cohort. The second and final follow-up was conducted 12 months after recruitment (±4 weeks) for all four cohorts. Follow-up assessments were conducted in a private space at the participants’ home. All assessments comprised a range of measures, including socio-demographic and economic measures, clinical severity measures, as well as healthcare expenditure measures, stigma and discrimination measures.^18^

Participants who could not be reached within the window period after at least three contact attempts were considered suspended until the next assessment, when an attempt to contact them was made again. The reason for suspending participants from follow-up was recorded. Participants who actively refused to be assessed at follow-up were withdrawn from the study. Suspensions from assessments or withdrawals from the study did not, however, affect the care participants in the treatment cohorts were receiving as part of the MHCP. Participants who, on the other hand, refused or discontinued treatment remained in the cohort and were still followed up for their assessments.

#### Outcomes

Primary outcomes for all cohorts was change in symptom severity, measured using the PHQ-9 (depression and maternal depression cohorts) and the AUDIT (AUD cohort). There was no symptom severity outcome for the psychosis cohort. Secondary outcomes for the depression cohort was response at each follow-up assessment, defined as a minimum 50% decrease in PHQ-9 score compared with baseline, as well as early remission (PHQ-9 <10 at 3 months), late remission (PHQ-9 <10 at 12 months but not at 3 months) and recovery (PHQ-9 <10 at both 3 and 12 months). Late remission was also a secondary outcome for the AUD cohort, defined as AUDIT <8 at 12 months. In addition to this, functioning at each follow-up, measured using the 12-item WHO Disability Assessment Schedule (WHODAS V.2.0) was included as a secondary outcome. A software malfunction prevented the measurement of the AUDIT among the AUD cohort at 3 months after recruitment. In the case of the psychosis cohort only functioning was assessed using WHODAS V.2.0.

#### Statistical analysis

None of the continuous outcome measures (eg, WHODAS, PHQ-9, AUDIT) were normally distributed, and so count regression models were used to assess these outcomes.^19 20^ More specifically, for the depression and AUD cohorts, mixed-effect negative binomial regression was used to assess difference-in-differences of outcome means at each time point in comparison to the baseline. Mixed-effect logistic regression was used to assess change in binary outcomes (response, early remission, late remission and recovery). Analysis was as per intention to treat and baseline imbalances in sociodemographic and clinical measures between the treatment and comparison groups in the depression and AUD cohorts were adjusted in the models. Non-parametric paired tests were conducted to assess change in PHQ-9 and/or WHODAS scores in the maternal depression and psychosis cohorts, due to the small sample sizes.

To assess equity in change in PHQ-9 (depression cohort) and AUDIT (AUD cohort) from baseline to 12 months across demographic groups, mixed effects negative binomial regression was performed again, this time including each demographic variable as an interaction term in the model; this was followed by Wald chi square tests. Equity in change in PHQ-9 scores (maternal depression cohort) and WHODAS scores (psychosis cohort) was assessed using non-parametric tests (Mann-Whitney U or Kruskal-Wallis tests).

## Results

### Community survey

#### Characteristics of the participants

In the baseline community survey, 3233 of 5170 randomly selected individuals were located (62.5%), and interviews were completed with 3220 participants. In the current analysis, we use data of 1305 participants who belonged to the PRIME implementation area (the baseline sample population for this paper). In the follow-up community survey, 2970 of 3898 randomly selected individuals were located (76.2%) and interviews were completed with 2867 participants (98.5%) which constitutes the follow-up survey population. Higher proportion of people in the baseline sample were younger, males, less educated and unemployed (table 1).

**Table 1:** Demographic and socioeconomic characteristics of the sample

	Community survey		Facility Detection Survey		Cohort study			
					Depression (n=409)*		Alcohol use disorder (n=339)†	
	Baseline (n=1305)	Follow-up (n=2867)	Baseline (n=760)	Follow-up (n=817)	Comparison (n=149)	Treatment (n=238)	Comparison (n=135)	Treatment (n=204)
Age (years)	P=0.026		P=0.006		P<0.001		P=0.880	
18–29	386 (29.8)	858 (30.0)	218 (26.7)	272 (33.3)	22 (15.3%)	48 (22.1%)	29 (21.5%)	42 (20.6%)
30–49	593 (45.2)	1210 (42.4)	324 (42.6)	285 (34.9)	53 (36.8%)	116 (53.5%)	72 (53.3%)	114 (55.9%)
50+	326 (25.1)	799 (27.6)	218 (28.7)	260 (31.8)	69 (47.9%)	53 (24.4%)	34 (25.2%)	48 (23.5%)
Gender	P<0.001		P=0.332		P<0.001		P>0.999	
Male	751 (57.5)	1455 (50.8)	374 (49.2)	422 (51.2)	100 (69.4%)	99 (45.6%)	135 (100.0%)	203 (99.5%)
Female	554 (42.5)	1412 (49.2)	386 (50.8)	395 (48.8)	44 (30.6%)	118 (54.4%)	0	1 (0.5%)
Religion	P=0.535		P<0.001		P=0.448		P=0.652	
Hindu	1187 (91.3)	2587 (89.3)	554 (72.9)	668 (81.7)	120 (83.3%)	188 (86.6%)	134 (99.3%)	200 (98.0%)
Muslim/Others	118 (8.7)	280 (10.7)	206 (27.1)	149 (18.2)	24 (16.7%)	29 (13.4%)	1 (0.7%)	4 (2.0%)
Marital status	P=0.539		P=0.036		P=0.005		P=0.216	
No partner	132 (10.2)	275 (9.4)	65 (8.6)	96 (11.8)	6 (4.2%)	6 (4.2%)	10 (7.4%)	8 (3.9%)
Has a partner	1173 (89.8)	2592 (90.6)	695 (91.4)	721 (88.2)	138 (95.8%)	138 (95.8%)	125 (92.6%)	196 (96.1%)
Education	P=0.013		P=0.017		P=0.142		P=0.907	
Uneducated/illiterate	773 (59.6)	1727 (60.4)	381 (50.1)	362 (44.3)	46 (31.9%)	79 (36.4%)	31 (23.0%)	48 (23.5%)
Less than primary education	434 (33.0)	830 (38.8)	186 (24.5)	250 (30.6)	47 (32.6%)	50 (23.0%)	40 (29.6%)	56 (27.5%)
Primary education or above	98 (7.4)	310 (10.7)	193 (25.4)	205 (25.1)	51 (35.4%)	88 (40.6%)	64 (47.4%)	100 (49.0%)
Employment status	P<0.001		P=0.032		P=0.005		P>0.999	
Unemployed	49 (3.6)	24 (0.8)	15 (2.0)	6 (0.7)	39 (27.1%)	91 (41.9%)	8 (5.9%)	12 (5.9%)
Employed	1256 (96.3)	2843 (99.2)	745 (98.0)	811 (99.3)	105 (72.9%)	126 (58.1%)	127 (94.1%)	192 (94.1%)

*Missing demographic data for 26 participants.

†Missing data for 26 participants.

#### Process data (community processes)

PRIME was implemented in three CHCs which provide services to 239 villages and cover a population of 268 833. PRIME Case Managers were able to visit and undertake community activities in only 136 villages (57.6% of total villages under three CHCs) which had a total population of 164 440 (approximately 98 664 adults assuming 60% of the total population is adult). They visited these 136 villages at least once during the implementation period. They screened 5540 individuals (5.6% of the total adult population) and referred 1428 (25.9%) to the facilities for further management. Among those who received the referral slips, 274 (19.2%) individuals came to the CHC to seek services and 189 (69%) of them were enrolled in the PRIME programme and received PRIME interventions.

#### Community level outcomes

The contact coverage in the baseline community survey was 14.8% (95% CI 11.1% to 19.4%) for depression and 7.7% (95% CI 2.8% to 19.5%) for AUD. There was no evidence of change in contact coverage for depression and AUD after implementation of PRIME MHCP. In the follow-up community survey, the contact coverage for depression was 10.5% (95% CI 7.6% to 14.3%) and for AUD it was 7.3% (95% CI 3.1% to 16.2%).

### Facility detection survey

#### Characteristics of the participants

In the baseline FDS, 760 randomly selected patients attending CHC outpatient clinic were contacted and interviews were completed with 760 (100%) participants (the baseline sample population). In the follow-up FDS, 920 randomly selected patients attending CHC outpatient clinic were contacted and interviews were completed with 817 (88.8%) participants (the follow-up sample population). Higher proportion of people in the baseline sample were younger, illiterate, didn’t have partner, unemployed and belonged to Muslim (or other) religion (table 1).

#### Process data (facility processes)

During the implementation period, a total of 131 884 outpatient clinic visits were recorded. Case Managers were able to screen 15 130 patients. Out of those screened, 1109 (7.3%) were enrolled to receive treatment for depression and 617 (4.1%) were enrolled to receive treatment for AUD.

#### Facility level outcomes

In the baseline FDS, none of the individuals attending outpatient clinic and who had depression or AUD were diagnosed with these conditions and therefore none of them received treatment. In the follow-up survey, 9.7% (95% CI 5.5% to 14.0%) of all the individuals who had depression and who were attending the outpatient department (OPD), were diagnosed with depression and were initiated on treatment. 19 participants were diagnosed with depression by medical officers, out of these 18 (94.7%) were true positive while 1 (5.3%) was false positive. 17.8% (95% CI 6.6% to 28.9%) of all the individuals who had AUD and who were attending the OPD, were diagnosed with AUD and were initiated on treatment (table 2).

**Table 2:** Impact of Programme for Improving Mental Health Care mental healthcare plan on community and facility level outcomes

	Depression		Alcohol use disorders	
	Baseline contact coverage (% with 95% CI)	Follow-up contact coverage (% with 95% CI)	Baseline contact coverage (% with 95% CI)	Follow-up contact coverage (% with 95% CI)
Community Survey	14.8 (11.1 to 19.4)	10.5 (7.6 to 14.3)	7.7 (2.8 to 19.5)	7.3 (3.1 to 16.2)

	Baseline detection coverage (% with 95% CI)	Follow-up detection coverage (% with 95% CI)	Baseline detection coverage (% with 95% CI)	Follow-up detection coverage (% with 95% CI)
Facility Detection Survey	0.0	9.7 (5.5 to 14.0)	0.0	17.8 (6.6 to 28.9)

### Cohort study

#### Depression

##### Characteristics of the participants

A total of 240 participants were recruited in the depression treatment cohort at baseline, and 149 participants in the comparison group. Two participants in the treatment cohort scored below 10 on the PHQ-9 at baseline, so they were excluded from the analysis. The demographic characteristics of the depression cohort’s final sample are reported in table 1. The proportion of females and those who reported not having a partner (single/divorced/widowed) was much greater in the depression treatment group compared with the comparison group. The patients in the treatment group were also much younger and unemployed (table 1). To adjust for these differences, all subsequent regression models controlled for gender, age, marital status and employment status. Baseline WHODAS and PHQ-9 scores were also different between the two groups, and so these were also included in the models.

**Figure 2:**
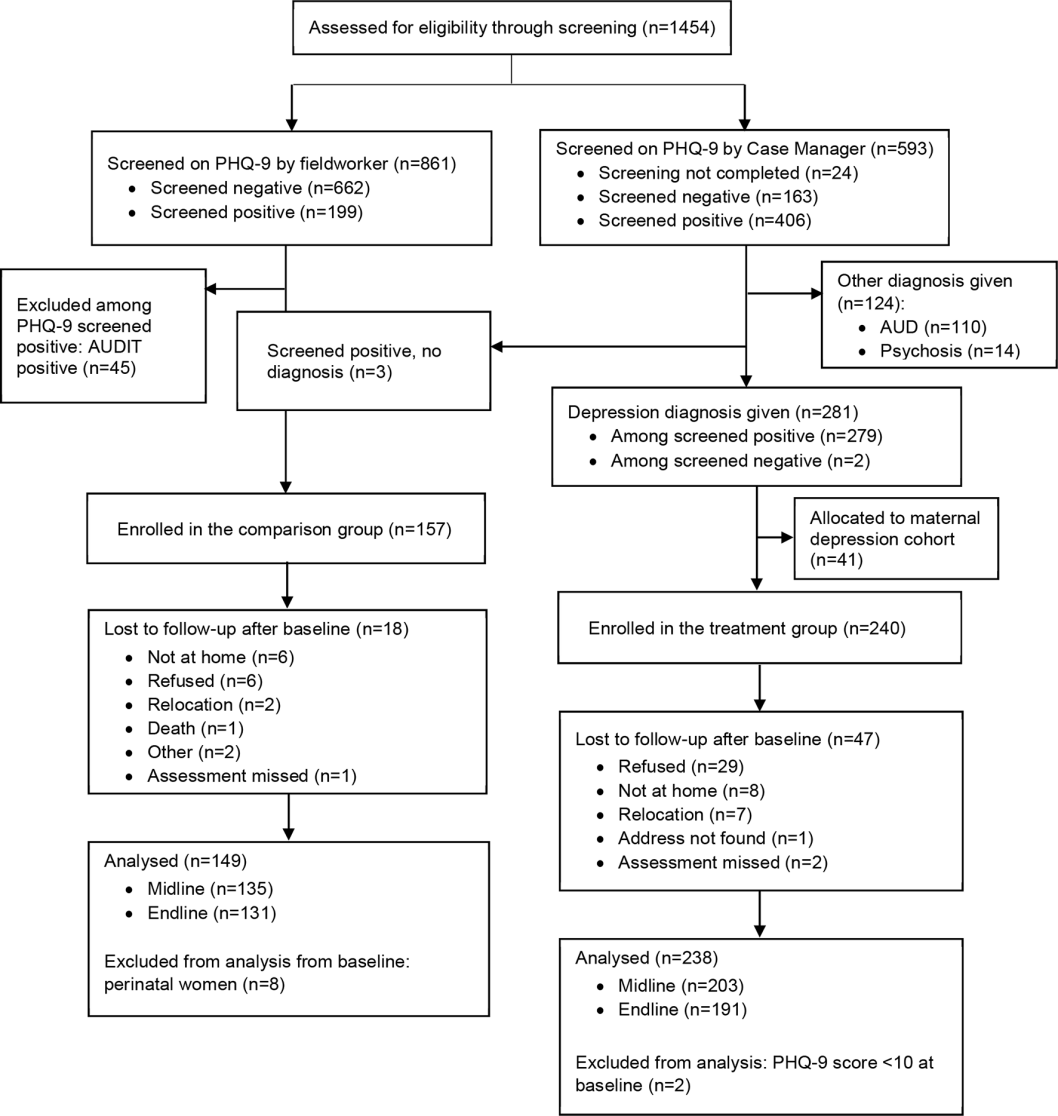
Flow diagram of recruitment and follow-up process for the depression cohort. PHQ-9, Patient Health Questionnaire-9 item.

The follow-up rates at 3 months were 90.6% and 85.3% for the comparison and treatment groups, respectively, and 87.9% and 80.3% at 12 months (figure 2).

A further 41 perinatal women were recruited into the depression cohort and analysed separately. All were married and nearly all were between the ages of 18 and 29 (n=38, 92.7%), Muslim (n=38, 92.7%), unemployed (n=39, 95.1%) but food secure (n=40, 97.6%). Over half reported having achieved primary education or above (n=27, 65.8%).

A total of 38 (92.7%) and 33 (80.5%) participants were followed up at 3 months and 12 months after recruitment, respectively.

##### Process data (depression and maternal depression cohort participants)

Of the 238 treatment participants, HAP was offered to all, and the first session of HAP was completed by 221 (92.9%) of participants. Only 34 participants (14.3%) completed four sessions of HAP. Treatment closure was done for 18 (7.6%) participants. Anti-depressants were prescribed to 177 participants (74%).

In case of perinatal women, first session of HAP was delivered to 40 participants (98%). However, it was very difficult to have follow-up sessions with them and only three participants (7.3%) received a second session of HAP.

##### Patient level outcomes

There was a significant difference in symptom severity reduction at 3 months between the intervention and comparison group of the depression cohort (table 3): participants in the intervention group had a mean PHQ-9 score of 14.5 (SD=3.22) at baseline, which decreased by 6.02 (95% CI −7.00 to 5.05) points at midline, whereas the participants in the comparison group saw their scores reduce by 3.18 points (95% CI −4.04 to 2.31), from a mean score of 13.4 (SD=2.63) at baseline. The difference in reduction in mean PHQ-9 score between the intervention and comparison group at midline (β=−2.85; 95% CI −3.88 to 1.81) and endline (β=−2.57; 95% CI −3.58 to −1.55) was clinically significant. This is also supported by difference in response and remission rates for depression.

**Table 3:** Impact of Programme for Improving Mental Health Care mental healthcare plan on individual level outcomes in the depression and AUD cohort

	Comparison group			Treatment group			Unadjusted β or RR (95% CI)	Adjusted β or RR (95% CI)
	N	Mean (SD) or N (%)	Adjusted mean change (95% CI) from BL	N	Mean (SD) or N (%)	Adjusted mean change (95% CI) from BL		
Depression cohort								
PHQ-9 score								
Baseline	149	13.4 (2.63)	–	238	14.5 (3.22)	–		
Midline	134	9.6 (4.18)	−3.18 (−4.04 to −2.31)	201	7.5 (3.92)	−6.02 (−7.00 to −5.05)	−3.22 (−4.352 to −2.09)	−2.85 (−3.88 to −1.81)***
Endline	131	9.0 (3.98)	−3.69 (−4.59 to −2.79)	191	7.3 (4.15)	−6.26 (−7.25 to −5.26)	−2.88 (−4.00 to −1.76)	−2.57 (−3.58 to −1.55)***
WHODAS score								
Baseline	144	30.7 (13.71)	–	217	26.4 (15.14)			
Midline	135	26.8 (13.59)	−3.52 (−6.08 to −0.97)	203	21.2 (11.22)	−4.07 (−5.89 to −2.25)	−0.69 (−4.44 to 3.06)	−0.55 (−3.50 to 2.41)
Endline	130	26.9 (12.87)	−3.61 (−6.18 to −1.04)	191	22.8 (11.93)	−2.82 (−4.61 to −1.04)	0.90 (−2.92 to 4.73)	0.79 (−2.22 to 3.80)
Response (PHQ-9)								
Midline	134	36 (26.9)	–	201	105 (52.2)	–	1.94 (1.43 to 2.65)	1.68 (1.22 to 2.30)**
Endline	131	40 (30.5)	–	191	109 (57.1)	–	1.87 (1.40 to 2.49)	1.56 (1.16 to 2.10)**
Early remission (PHQ-9)	134	60 (44.8)	–	201	141 (70.2)	–	1.57 (1.27 to 1.93)	1.50 (1.21 to 1.87)***
Late remission (PHQ-9)	73	31 (42.5)	–	57	29 (50.9)	–	1.20 (0.83 to 1.73)	1.24 (0.84 to 1.84)
Recovery (PHQ-9)	130	37 (28.5)	–	189	106 (56.1)	–	1.97 (1.46 to 2.66)	1.82 (1.33 to 2.48)***
AUD Cohort								
AUDIT Score								
Baseline	147	14.3 (4.88)	–	217	18.4 (7.76)	–	–	–
Endline	104	10.4 (5.44)	−3.32 (−4.47 to −2.17)	137	11.3 (7.48)	−6.03 (−7.21 to −4.85)	−3.23 (−5.14 to −1.33)	−2.71 (−4.29 to −1.13)**
WHDOAS score								
Baseline	135	22.4 (11.73)		204	19.4 (12.80)			
Midline	128	21.7 (10.23)	−0.54 (−2.94 to 1.86)	190	18.2 (10.64)	−0.66 (−2.20 to 0.89)	−0.33 (−3.64 to 2.98)	0.12 (−2.97 to 2.74)
Endline	118	22.2 (12.41)	−0.58 (−3.05 to 1.88)	174	19.4 (10.21)	0.56 (−1.08 to 2.20)	1.26 (−2.17 to 4.70)	1.14 (−1.82 to 4.10)
Late remission (AUDIT)	104	25 (24.0)	–	137	43 (31.4)	–	1.31 (0.86 to 1.99)	1.44 (0.95 to 2.20)

*P<0.05; **p<0.01; ***p<0.001.

AUD, alcohol use disorder; AUDIT, Alcohol Use Disorder Identification Test;BL, Baseline;PHQ-9, Patient Health Questionnaire-9 item;RR, risk ratio; WHODAS, WHO Disability Assessment Schedule.

Response on the PHQ-9 at 3 months was significantly higher in the treatment group (52.2%) compared with the comparison group (26.9%) (adjusted risk ratio (aRR)=1.68; 95% CI 1.22 to 2.30). The difference was still significant at 12 months (aRR=1.56; 95% CI 1.16 to 2.10). The proportion of participants reporting early remission (PHQ-9 <10 at 3 months) on the PHQ-9 was greater among the treatment participants (70.2%) compared with the comparison group (44.8%), (aRR=1.50; 95% CI 1.21 to 1.87). Recovery from depression (defined as PHQ-9 <10 at both 3 and 12 months) was also significantly higher in the treatment group (56.1%) compared with the comparison group (28.5%) (aRR=1.82; 95% CI 1.33 to 2.48). There was no difference in late remission of depression in the treatment group compared with the comparison group. However, improvement in functioning was not different between the two groups at 3 months (β=−0.55; 95% CI −3.50 to 2.41) or 12 months (β=0.79; 95% CI −2.22 to 3.80). There was no evidence of non-equity in change in PHQ-9 scores at 12 months across demographic groups (table 4).

**Table 4:** Depression cohort: multivariable association of demographic and socio-economic factors with change in PHQ-9 score at 12 months

	Comparison group			Treatment group			Difference of difference (95% CI)	Wald χ^2^
	N	Mean (SD)	Adjusted mean change (95% CI) from BL	N	Mean (SD)	Adjusted mean change (95% CI) from BL		
Gender								
Male	88	8.8 (4.09)	−4.09 (−5.19 to −2.98)	85	7.1 (4.29)	−6.36 (−7.65 to −5.06)	−2.27 (−3.63 to −0.92)	1.58
Female	43	9.5 (3.74)	−3.21 (−4.67 to −1.75)	105	7.4 (4.06)	−6.75 (−7.91 to −5.60)	−3.55 (−5.26 to −1.83)	
Age (years)								
18–29	19	7.9 (4.68)	−5.19 (−7.39 to −3.00)	39	6.0 (3.73)	−8.11 (−9.91 to −6.31)	−2.92 (−5.48 to −0.35)	3.94
30–49	49	8.2 (3.99)	−4.46 (−5.92 to −3.01)	99	7.3 (4.33)	−7.07 (−8.40 to −5.73)	−2.60 (−4.24 to −0.97)	
50+	63	10.0 (3.53)	−3.56 (−4.87 to −2.24)	52	8.2 (3.93)	−5.66 (−7.15 to 4.17)	−2.10 (−3.91 to −0.29)	
Religion								
Hindu	109	8.9 (4.10)	−3.83 (−4.83 to −2.82)	169	7.1 (4.26)	−6.43 (−7.57 to −5.28)	−2.60 (−3.72 to −1.48)	0.51
Other	22	9.6 (3.32)	−3.19 (−5.09 to −1.28)	21	8.4 (3.02)	−5.35 (−7.32 to −3.39)	−2.17 (−4.80 to 0.46)	
Education								
Uneducated/Illiterate	41	9.4 (3.48)	−3.32 (−4.70 to −1.94)	71	7.8 (4.42)	−6.15 (−7.47 to −4.84)	−2.83 (−4.52 to −1.14)	3.13
Less than primary education	43	9.7 (4.03)	−3.26 (−4.67 to −1.85)	47	7.9 (4.15)	−5.42 (−6.88 to −3.96)	−2.16 (−4.03 to −0.29)	
Primary education or above	47	8.1 (4.24)	−4.58 (−5.98 to −3.18)	72	6.4 (3.78)	−7.04 (−8.345 to −5.74)	−2.46 (−4.09 to −0.83)	
Marital status								
No partner	6	8.2 (5.91)	−3.74 (−7.05 to −0.44)	23	8.7 (3.99)	−5.85 (−7.71 to −3.99)	−2.11 (−5.81 to 1.59)	0.09
Has a partner	125	9.1 (3.89)	−3.44 (−4.27 to −2.61)	167	7.1 (4.15)	−5.90 (−6.85 to −4.95)	−2.47 (−3.47 to −1.47)	
Occupation								
Unemployed	39	9.5 (3.95)	−3.22 (−4.71 to −1.73)	80	7.0 (4.00)	−6.88 (−8.25 to −5.510)	−3.66 (−5.46 to −1.86)	0.85
Employed	92	8.9 (3.99)	−4.07 (−5.11 to −3.02)	110	7.5 (4.27)	−6.14 (−7.24 to −5.03)	−2.07 (−3.34 to −0.80)	
Food insecurity								
Food secure	123	9.0 (4.06)	−4.01 (−4.96 to −3.06)	182	7.2 (4.11)	−6.60 (−7.61 to −5.59)	−2.59 (−3.67 to −1.50)	1.83
Food insecure	8	10.3 (2.25)	−1.73 (−4.90 to 1.45)	8	9.8 (4.59)	−5.92 (−9.52 to −2.32)	−4.19 (−8.96 to 0.58)	

BL, Baseline;PHQ-9, Patient Health Questionnaire-9 item.

Symptom severity decreased significantly over time among participants in the maternal depression cohort: the non-parametric paired tests showed a mean decrease in PHQ-9 scores of 7.50 points (95% CI −8.72 to −6.28; Z=−5.26, p<0.001), from baseline to 3 months, and a reduction of 8.4 points 95% CI −9.50 to −7.29; −5.02; p<0.001) from baseline to 12 months. The reduction in WHODAS scores was significant at 12 months (mean=−7.66; 95% CI −12.84 to −2.48; Z=−2.40; p=0.017), but not at 3 months (mean=−2.12; 95% CI −7.48 to 3.24; Z=−0.21, p=0.833). Also, 76.3% showed a response on the PHQ-9 at midline (95% CI 59.84% to 87.45%) and 78.8% (95% CI 61.06% to 89.80%) at endline. As in the depression cohort, there were no signs of inequity in reduction in PHQ-9 scores at 12 months across demographic groups.

### Alcohol use disorders

#### Characteristics of the participants

In the AUD cohort at baseline, 218 participants were recruited in the treatment group and 147 patients in the comparison group. Only one participant in the treatment group screened negative on the AUDIT and was excluded from the analysis; the final sample for the treatment group was 217. All but one of the participants recruited in the AUD cohort were males. The demographic and socio-economic characteristics were otherwise similar for both groups (table 1). Baseline WHODAS and AUDIT scores did differ between the two groups, and so both scores were controlled for in subsequent analyses.

**Figure 3:**
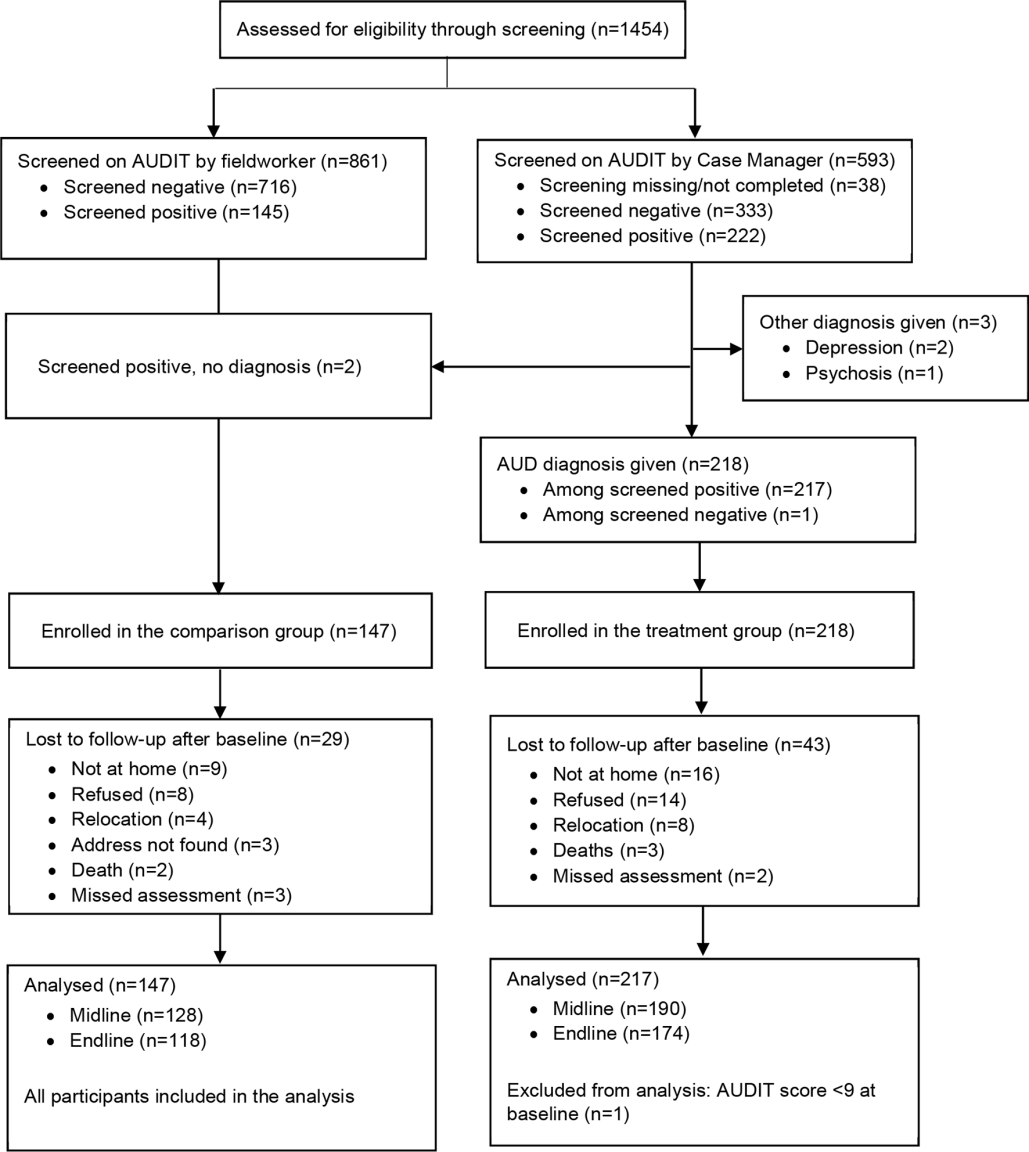
Flow diagram of recruitment and follow-up process for the AUD cohort. AUD, alcohol use disorder; AUDIT, Alcohol Use Disorder Identification Test.

Follow-up rates at 3 months were 87.1% and 87.6% for the comparison and treatment groups, respectively, and 80.3% and 80.2% at 12 months (figure 3).

#### Process data (AUD cohort participants)

Out of 217 participants enrolled in the AUD cohort, 203 (93.6%) were delivered the first session of CAP. Only 12 participants (5.5%) completed four sessions of CAP. Treatment closure was done in 34 (15.7%) participants. Multi-vitamins were prescribed to 143 (65.9%) participants. Thiamine was not prescribed as it was not available in CHCs.

#### Patient level outcomes

Change in symptom severity from baseline to 12 months was significantly different between the intervention and comparison groups in the AUD cohort (table 3): scores decreased by 6.0 points (95% CI −7.21, to 4.85) in the intervention group, compared with a reduction of 3.3 points (95% CI −4.47 to 2.17) in the comparison group (β=−2.71; 95% CI −4.29 to −1.13). The improvement in functioning from baseline was not different between the two groups, whether at 3 months or 12 months after recruitment (table 3). The proportion of participants showing late remission, (AUDIT <8 at 12 months) was also not significantly different between the treatment group and the comparison group.

Table 5 presents the change in AUDIT scores from baseline to 12 months in the AUD cohort across demographic groups. Results of the Wald test suggest that the reduction in scores was greatest among those reporting no education and among those reporting more than primary education (Wald χ^2^=6.66; p=0.036), participants who had completed primary education (or above) had higher reduction in AUDIT scores. The reduction in AUDIT scores at 12 months was also marginally greater among younger participants (Wald χ^2^=3.94, p=0.083).

**Table 5:** AUD cohort: multivariable association of demographic and socio-economic factors with change in audit score at 12 months

	Comparison group			Treatment group			Difference of difference (95% CI)	Wald χ^2^
	N	Mean (SD)	Adjusted mean change (95% CI) from BLb	N	Mean (SD)	Adjusted mean change (95% CI) from BLb		
Gender								
Male	104	10.4 (5.44)	−3.31 (−4.44 to −2.18)	136	11.4 (7.45)	−5.95 (−7.12 to −4.78)	−2.65 (−4.21 to −1.08)	–
Female	0	–	–	1	0.0 (−)	−10.92 (−21.20 to −0.63)	–	
Age (years)								
18–29	22	11.4 (4.63)	−1.57 (−4.03 to 0.90)	25	11.2 (8.26)	−5.40 (−7.84 to −2.96)	−3.84 (−7.30 to −0.37)	**4.98** m
30–49	56	10.6 (5.27)	−3.33 (−4.88 to −1.77)	81	12.4 (7.30)	−5.81 (−7.36 to −4.26)	−2.49 (−4.63 to −0.35)	
50+	26	9.0 (6.29)	−4.60 (−6.66 to −2.55)	31	8.5 (6.81)	−7.08 (−9.19 to −4.96)	−2.47 (−5.32 to 0.37)	
Religion								
Hindu	103	10.4 (5.46)	−3.33 (−4.48 to −2.18)	135	11.3 (7.38)	−5.96 (−7.14 to −4.78)	−2.63 (−4.22 to −1.04)	0.04
Other	1	9.0 (−)	−1.62 (−11.73 to 8.49)	2	12.5 (17.68)	−9.57 (−18.89 to −0.26)	−7.96 (−21.70 to 5.78)	
Education								
Uneducated/Illiterate	22	7.2 (4.83)	−4.943 (−6.97 to −2.90)	29	9.2 (7.08)	−7.70 (−9.91 to −5.49)	−2.76 (−5.70 to 0.173)	6.66*
Less than primary education	34	10.7 (5.50)	−3.34 (−5.30 to −1.38)	42	11.3 (6.67)	−4.07 (−5.97 to −2.17)	−0.73 (−3.436 to 1.97)	
Primary education or above	48	11.60 (5.17)	−2.47 (−4.15 to −0.79)	66	12.2 (8.05)	−6.33 (−7.98 to −4.67)	−3.86 (−6.19 to −1.52)	
Marital status								
No partner	7	10.0 (6.78)	−4.85 (−8.90 to −0.79)	6	8.3 (8.24)	−3.27 (−7.46 to 0.93)	1.58 (−4.24 to 7.40)	0.70
Has a partner	97	10.4 (5.37)	−3.21 (−4.40 to −2.02)	131	11.4 (7.45)	−6.15 (−7.37 to −4.94)	−2.94 (−4.58 to −1.31)	
Occupation								
Unemployed	7	10.0 (5.07)	−2.49 (−6.72 to 1.74)	8	9.9 (12.48)	−9.24 (−13.90 to −4.59)	−6.76 (−13.01 to −0.51)	0.10
Employed	97	10.4 (5.49)	−3.39 (−4.57 to −2.21)	129	11.4 (7.13)	−5.85 (−7.04 to −4.65)	−2.46 (−4.08 to −0.84)	
Food insecurity								
Food secure	97	10.5 (5.37)	−3.25 (−4.45 to −2.06)	126	10.9 (7.48)	−6.20 (−7.43 to −4.97)	−2.95 (−4.59 to −1.30)	0.65
Food insecure	7	8.9 (6.52)	−4.70 (−8.97 to −0.42)	11	15.6 (6.25)	−5.06 (−9.68 to −0.45)	−0.37 (−6.58 to 5.85)	

*P<0.05.

AUD, alcohol use disorder; AUDIT, Alcohol Use Disorder Identification Test;BL, Baseline;m, marginal.

### Psychosis

#### Characteristics of the participants

Finally, 22 participants were recruited in the psychosis cohort. Demographic information was available for 20 of these participants, and the majority were between 30 and 49 years of age (n=11, 55.0%), were male (n=13, 65.0%), Hindu (n=18, 90.0%), married (n=14, 70.0%) and report achieving primary education or above (n=12, 60.0%). Half of the participants were unemployed, and all but one reported being food secure (n=19, 95%). Eighteen participants (81.8%) were followed up at 3 months and 12 months. We were unable to recruit the expected number of psychosis patients (N=150) in the cohort study as most of the patients with psychosis in the implementation area accessed care from psychiatrists in Bhopal.

#### Process data (psychosis cohort participants)

Medications (anti-psychotics) were prescribed to 18 out of 22 (81.8%) participants and 20 participants (90.9%) were provided psychoeducation. Three follow-up sessions were completed by six participants (27.3 %).

#### Patient level outcomes

The non-parametric paired test suggests that the reduction in WHODAS scores among the participants recruited in the psychosis cohort was not significant, either at 3 months (β=−3.40; 95% CI −10.85 to 4.06; Z=−1.11; p=0.266) or 12 months (β=−6.25; 95% CI −14.74 to 2.24; Z=−1.63; p=0.103).

## Discussion

We report the findings of the impact evaluation of PRIME, India, a multiplatform, multicomponent mental health programme, which aimed to integrate mental health services in primary care in Sehore district of Madhya Pradesh. PRIME MHCP did not have any impact on contact coverage for depression and AUD at the community level and a small impact on detection and initiation of treatment for depression and AUD in the health facilities. This seems to indicate that screening by PRIME Case Managers and training of medical officers had only a small impact on detection and treatment initiation. At the individual patient level, there was moderate impact of MHCP interventions. Patients with depression who received care as part of the MHCP had higher rates of response, early remission and recovery compared with those who did not receive care, but there was no impact of treatment on their functioning. It is difficult to attribute this impact to PRIME both due to the very low coverage of the evidence-based interventions as well as very large baseline differences between the treatment and comparison cohorts. Thus, at district level, PRIME MHCP had no impact on improving the coverage of services at population/community level, small impact on the health systems/facility level outcomes and modest impact on patient/individual level outcomes. In the section below, we try to explain these findings.

### Facility versus community focus

PRIME MHCP was designed to be implemented on the healthcare and community platforms and the emphasis was also to establish enabling packages to strengthen the health system. The Department of Health Services was primarily responsible for the delivery of mental health services while the role of the PRIME team was to be a facilitator and support team.^6^ Most of the government health programme including the District Mental Health Program are healthcare platform centred as the priority of the government is to first establish the services in the ‘formal’ healthcare system using the infrastructure under their authority. Services are generally initiated in the District Hospital first, followed by Sub-District Hospitals/CHCs and then in primary healthcare centres (PHCs) (when the Government of Madhya Pradesh decided to scale up mental health services, ‘Mann-Kaksha’ were first established in the District Hospitals, this is described below). The unit of implementation in the original PRIME proposal was PHC, but in India it was changed to CHC based on inputs from the key stakeholders and to generally align PRIME work with the existing approach of the government. As a result, facility processes on the healthcare platform took precedence over community processes and the Case Managers predominantly spent their time in CHCs to establish and optimise facility processes.

### Retention in care

The biggest challenge in MHCP implementation was our inability to retain patients with depression and AUD in continuing care. Only 12.3% of patients with depression received four sessions of HAP while 5.5% of patients with AUD received four sessions of CAP. The treatment was generally acceptable to patients, but their willingness to come to the CHC for follow-up sessions was minimal. One possible option to improve retention in care was by providing home-based HAP/CAP sessions. Unfortunately, provision of home-based therapy was not feasible in PRIME as Case Managers spent almost two-thirds of their time in the CHC. On other hand, we were unable to recruit additional human resources (eg, counsellors) through PRIME or the public health system to deliver home-based sessions of psychological interventions. In the PREMIUM trial which successfully demonstrated the effectiveness of HAP, 77% of the first sessions (typically on the day when patients were enrolled) were delivered in the PHC while 91% of the subsequent sessions were delivered at home.^9^ With hindsight, we feel that inability to plan home-based delivery of care was one of the major lacunae in programme design. Other possible options could have been involvement of CHWs such as ASHA in delivery of community interventions and follow-up care. Initially, efforts were made to involve ASHAs to screen patients in the community, provide MHFA, refer them to CHC and then provide follow-up care. ASHA workers receive monetary incentives mainly from the Maternal and Child Health programs to provide community-based care. We could not meaningfully engage ASHA workers for provision of psychological care as funds were not available either with PRIME or with the Government to give them incentives. This resulted in very low dosage of psychological intervention and should be considered as another major limitation of MHCP implementation. It must also be noted that around 1800 patients were enrolled in the programme while the cohort assessment only includes 400+ patients. In a trial, generally there are far more counsellors and number of patients per counsellor is far less as compared with number of patients who were provided counselling by PRIME Case Managers. Another important consideration is that the trial is a time-bound process while PRIME implementation was designed as an ongoing activity and even after the cohort enrolment was stopped, new patients were getting enrolled in the programme and had to be followed up. The PRIME evaluation therefore represents an important real-world assessment of routine integration of mental healthcare into the primary care system. In addition to provision of HAP and CAP, Case Managers also had to spend time on activities related to enabling packages (eg, HMIS, drug procurement) and interventions on community platform.

### Limited participation of the health system staff

PRIME Implementation at CHC level helped in reducing the ‘missed opportunity’^21^ to identify and treat patients attending outpatient clinics. The improvement in detection and initiation of treatment for depression and AUD was primarily due to screening by Case Managers and it was very modest, from 0% to 9.7% and 17.8%, respectively. Under-detection and inability to treat depression and AUD in primary care is due to multiple factors which can be broadly classified into three categories; patient characteristics and preferences, provider knowledge and attitudes and health system related factors.^22–25^ Patients attending outpatient clinics were not primarily seeking care for mental health problems but for other physical ailments. Another important barrier is shorter consultation times in primary care. A recently published systematic review found that in 18 countries (which represent 50% of the global population), patients spent less than 5 min with their primary care physicians. In India the average consultation time was 2 min.^26^ In our setting as well, medical officers had very limited time for patient consultation, other CHC staff (eg, nurse) did not proactively refer or detect patients ultimately resulting in only Case Managers dealing with screening and detection of depression and AUD. Due to time constraints, Case Managers could screen very limited numbers of patients attending outpatient clinics leading to minimal improvements in this particular outcome.

### Community outreach

PRIME implementation did not lead to any change in access to mental healthcare at the community level. This can be primarily explained by limited delivery of community-based interventions by Case Managers. As the process data indicates, very few individuals in the community were screened by Case Managers and were provided psychological first aid. Case Managers established linkages with CHWs such as Anganwadi workers and ASHA workers, but beyond facilitating visits of Case Managers the contribution of CHWs to screening and referral of individuals with depression, AUD and psychosis was minimal. One of the important reasons for this was lack of any incentive for CHWs to undertake these activities. VISHRAM (the Vidarbha Stress and Health Programme), a grass-roots community-based programme led by a team of CHWs and lay counsellors working in collaboration with primary care physicians and visiting psychiatrists led to a sixfold increase in contact coverage for depression.^27^ This could be achieved due to a dedicated CHW in each of the 30 implementation villages as well as a range of community interventions undertaken by CHWs to improve mental health literacy. CHWs were paid an honorarium of Rs. 1500 per month from the project funds. PRIME was implemented with far less human resource who had different set of responsibilities (primarily facility processes as explained above) and also had to cover a larger geographical area (PRIME community survey was done in 188 villages while VISHRAM survey and implementation was restricted to 30 villages). As mentioned earlier, in PRIME there was no provision to pay monetary incentives to ASHA workers to undertake community level interventions.

### Limitations

There are several limitations to this study. The allocation of patients with depression/AUD to treatment/comparison group was not random, which prevents us from drawing a causal inference about treatment–outcome relationship. In addition to this, Case Managers proactively screened patients with depression/AUD and offered them treatment. They contacted those patients in OPD who were more likely to have depression/AUD. As a result, patients with higher mean PHQ-9/AUDIT scores were enrolled in treatment group. It is possible that higher change observed in mean scores in treatment arms of both the cohorts is due to ‘regression to the mean’ phenomenon.^28^ We have not included correlates of change in treatment outcomes in our analyses, including the role of retention and number of sessions received and this could be the focus of future research. Screening tools (PHQ-9 and AUDIT) were used to identify probable cases of depression and AUD which increases the risk of misclassification of these disorders. We used the Hindi versions of PHQ-9 and AUDIT used extensively in the PREMIUM trials.^9 10^ These tools were not further validated for the Hindi-speaking population of Sehore district, Madhya Pradesh. We were able to recruit only 22 patients in psychosis cohort, as most of them accessed care from psychiatrists in Bhopal. In FDS, outcome ascertainment was based on patient exit interviews only without simultaneous analysis of clinical notes. In CS, we were unable to contact 37.5% (baseline) and 23.8% (follow-up) of the individuals sampled from the voter list which might have resulted in selection bias.

### Lessons learnt

Evaluation findings demonstrate minimal impact of PRIME MHCP on key primary outcomes, however, there are important lessons from the implementation of the MHCP which can be helpful in future integration of mental health services in primary care in low resource settings.

The key learnings are (1) mapping of service delivery processes on healthcare and community platform are helpful in monitoring/tracking the progress and identifying barriers in service delivery; (2) facilitation by an implementation support team can address some of these barriers; (3) enabling packages of MHCP play a crucial role in strengthening the health system and improving the context and settings for implementation; (4) engagement with key community stakeholders and incentives for CHWs (ASHAs in case of India) are necessary to deliver services on the community platform.

### Sustainability and implications for mental health practice

In 2015, Department of Health Services decided to scale up the PRIME model across 51 District Hospitals in Madhya Pradesh.^29^ As part of this initiative, ‘Mann-Kaksha’ were established in District Hospitals and a minimum of two nurses and one medical officer from each district were trained to provide mental health services. The suggestion to appoint Case Managers in District Hospitals was not accepted, but it was decided that nurses will fulfil the functions of Case Managers. Very recently, operationalisation of ‘Mann-Kaksha’ to strengthen mental health services was awarded as a best practice by the National Health Mission.^30^ The adoption of the PRIME Implementation model by the Government of Madhya Pradesh for state-wide scale up of mental health services ensures the sustainability of the work initiated by PRIME.

## Conclusions

Establishing a collaborative model of care remains a very challenging task, especially in resource poor settings. Facilitation by an implementation support team can play an important role in establishing enabling packages related to governance, health information systems and so on which serve as foundation to instal, optimise and improve service delivery processes. Training of primary care physicians alone is not a sufficient intervention to improve diagnosis and provision of minimally adequate treatment for priority mental disorders. Routine screening of these disorders and inclusion of quality improvement strategies to improve various care processes must be included in future implementation of mental health programme. Our major learning is that psychosocial therapies cannot be effectively delivered without engaging existing or new community resources. While dedicated human resources (eg, Case Managers) and dedicated space for mental health clinics (eg, Mann-Kaksha) strengthen the facility/healthcare platform, CHWs such as ASHAs could be potentially trained to deliver psychosocial interventions in the community ensuring delivery of the adequate dosage of the intervention. A crucial lesson is that without substantial additional investments in staff, such as CHWs/ASHAs to improve community level case detection and conduct home visits to deliver the HAP and CAP psychological treatments, we are unlikely to see major changes in coverage or clinical outcomes. One of the key actions recommended by the recently launched Lancet Commission on global mental health and sustainable development^31^ is substantial additional investment in mental health and redistribution of mental health budgets from large hospitals to District Hospital and community-­based local services. Strong advocacy efforts are needed at national and provincial level to implement these recommendations by allocating resources to recruit and train community health workers to deliver mental healthcare in the community. It is also important to recognise that mental health programme must be both bottom up and top-down (ie, community driven and healthcare facility driven, and addressing both supply-side and demand-side factors). In addition to the primary healthcare system, local communities should be involved in mental health programme as it was done in the VISHRAM programme.^27^ A synthesis of learnings from PRIME and VISHRAM model can potentially inform scaling up of mental health services across India through the National Mental Health Programme.
